# From Formula Composition to Precision Nutrition: Who Benefits, From What, and When?

**DOI:** 10.1097/CCE.0000000000001497

**Published:** 2026-10-01

**Authors:** Sue Brierley-Hobson, Ashley DePriest

**Affiliations:** 1 Betsi Cadwaladr University Health Board, NHS Wales, Bangor, United Kingdom.; 2 Bangor University, Bangor, United Kingdom.; 3 Grady Health System, Atlanta, GA.

**Keywords:** critical illness, enteral nutrition, food composition, formula composition, nutritional support, precision nutrition

## Abstract

Morowitz et al argue that the composition of enteral nutrition formulas deserves to be studied as an intervention in its own right. Building on this perspective, we consider how critical care nutrition research has progressively refined its questions from whether to feed to when and how much. We argue that enteral formula composition represents the next logical frontier in critical care nutrition research, while emphasizing the need for biologically informed, clinically meaningful, and practical interventions. We also highlight the importance of learning from previous translational challenges to ensure that future research identifies formulations that improve patient outcomes while remaining feasible for routine critical care practice.

**KEY POINTS**

- Critical care nutrition research has progressively moved from establishing whether and how to feed toward refining questions of timing and nutrient dose; formula composition represents a logical next dimension for investigation.
- Enteral formulas should not necessarily be regarded as biologically interchangeable vehicles for nutrient delivery; nutrients may also act as biological signals influencing inflammatory, neuroimmune, and gastrointestinal pathways.
- Mechanistic plausibility alone is insufficient: previous nutrition interventions with compelling biological rationales have often failed to translate into consistent clinical benefit, particularly within the heterogeneous biological environment of critical illness.
- Future formula-composition research should integrate mechanistic and clinical science while accounting for patient heterogeneity and real-world formulation constraints, ultimately prioritizing reproducible patient-important outcomes, safety, and feasibility.

We welcome the commentary by Morowitz et al (1) in the August 2026 issue of *Critical Care Explorations*, which argues that the composition of enteral nutrition formulas deserves to be studied as an intervention in its own right. Their central premise, that the critical care community has focused predominantly on the quantity and timing of nutritional delivery while paying comparatively little attention to formula composition, highlights an underexplored research question. We agree that understanding what we feed may represent the next major advance in critical care nutrition research.

Importantly, we also welcome the fact that these questions are being championed by the wider multidisciplinary critical care community. Dietitians have debated many aspects of formula composition for years, but broader engagement across critical care medicine may provide the momentum for a new generation of nutrition research. In this respect, we believe the commentary exemplifies one of the strengths of *Critical Care Explorations*: providing a forum for hypothesis-generating work that challenges existing paradigms and stimulates further scientific inquiry. The value of this commentary lies in articulating a question that the field may not yet have asked explicitly enough.

## REFRAMING THE EVIDENCE

The authors’ argument becomes even stronger when considered within the broader evolution of critical care nutrition research. The landmark trials cited by the authors, such as the Early vs. Delayed Enteral Nutrition (EDEN) trial and The Augmented vs. Routine Approach to Giving Energy Trial (TARGET) were designed principally to compare alternative strategies of nutrient delivery, asking whether differences in timing or energy provision translated into improved patient outcomes (2, 3). Rather than indicating that nutritional therapy is ineffective, these studies substantially advanced understanding of which nutritional strategies produce equivalent outcomes, which should be avoided, and where genuine uncertainty remains. Recent reviews suggest these apparently neutral findings should also be interpreted within their biological context, recognizing that many interventions were initiated during the acute phase of critical illness, when anabolic resistance may limit responsiveness to nutritional therapy (4). Collectively, these landmark trials have refined the research agenda, naturally leading to questions regarding additional dimensions of nutritional therapy, including formula composition.

We suggest the nutrition-outcome relationship may be elusive not because nutritional therapy lacks biological importance, but because successive generations of trials have asked progressively more focused questions within an extraordinarily heterogeneous patient population.

## THE EVOLUTION OF CRITICAL CARE NUTRITION RESEARCH

This progression becomes particularly clear when viewed historically. Early work established the importance of preventing malnutrition while defining the safest route of nutrient delivery, ultimately leading to enteral nutrition becoming the preferred approach when the gastrointestinal tract is functional. Subsequent decades focused on when and how much nutrition should be delivered, with landmark trials demonstrating that many competing feeding strategies resulted in similar clinical outcomes while refining modern practice.

Against this broader historical backdrop, understanding whether formula composition contributes meaningfully to patient response represents the logical next step, and the commentary by Morowitz et al (1) naturally extends this trajectory by posing the question made possible by decades of previous investigation.

## FROM NUTRIENT DELIVERY TO NUTRIENT SIGNALING

Implicit within many previous randomized nutrition trials has been the assumption that commercially available enteral formulas are biologically interchangeable, functioning primarily as vehicles for nutrient delivery rather than interventions with distinct biological properties. However, alongside large trials examining timing and dose, parallel mechanistic studies have increasingly challenged this assumption, suggesting that nutrients should be viewed as biological signals capable of influencing inflammatory, neuroimmune, and barrier function, supporting the authors’ premise that nutrients may exert biological effects beyond their role as caloric substrates. One particularly interesting example comes from studies in animal postoperative ileus and controlled human endotoxemia models, where lipid-enriched enteral nutrition has been shown to activate cholecystokinin (CCK)-mediated vagal anti-inflammatory pathways, attenuating inflammatory signaling and improving gastrointestinal motility or barrier-related outcomes (5, 6). Observational studies in critically ill patients have subsequently demonstrated changes in CCK, acetylcholine, and inflammatory biomarkers consistent with activation of similar neuroimmune pathways during early enteral nutrition (7), providing biological plausibility for the hypothesis advanced by Morowitz et al (1) that formula composition itself may represent a therapeutically relevant intervention.

The history of critical care nutrition research nevertheless reminds us that while mechanistic plausibility has repeatedly generated compelling hypotheses, subsequent studies have often failed to establish clinical benefit or justify changes in clinical practice. Numerous interventions, including immune-modulating formulas, glutamine, and antioxidant therapies such as vitamin C supplementation, were supported by intriguing mechanistic rationale and encouraging physiologic or clinical signals. However, subsequent large clinical trials and meta-analyses have produced mixed or inconsistent findings.

Together, these considerations illustrate the complexity of translating promising biological effects into reproducible improvements in patient outcomes. A further consideration is the biological environment in which enteral nutrition is delivered. As Morowitz et al (1) suggest, the gastrointestinal tract is not merely a route for nutrient delivery but an active interface between nutritional substrates, the microbiome, host immunity, and the external environment. During critical illness, interacting inflammatory, barrier, microcirculatory, and neuroimmune disturbances may alter both gastrointestinal function and the biological context in which nutrients exert their effects (8, 9). Further, the gastrointestinal environment into which an enteral formula is delivered is itself heterogeneous between patients, with differences in gut microbial diversity apparent at the point of ICU admission (10). Feeding intolerance and impaired motility may additionally limit the reliable delivery of those substrates. The challenge is therefore not simply to identify which nutritional substrates are biologically active, but to determine how their effects vary within this complex host environment and how they can be delivered safely and effectively.

With this in mind, as Morowitz et al (1) appropriately acknowledge, the inclusion of certain ingredients in contemporary enteral formulas reflects considerations beyond nutritional composition alone, with formula design already balancing complex physiologic requirements with safety, manufacturability, stability, osmolality, and microbial risk. For example, carbohydrate sources are often selected to optimize digestibility, osmolality, and intestinal absorption, while emulsifiers, stabilizers, and preservatives contribute to product stability, sterility, and shelf life. These design features are particularly important in critically ill and immunocompromised patients, whose vulnerability to infection and other complications may magnify the consequences of microbial contamination or formula instability.

Similar considerations apply to increasing the proportion of whole-food ingredients within enteral formulas. Although conceptually attractive, greater incorporation of minimally processed ingredients introduces additional challenges related to microbial safety, product consistency, nutrient stability, viscosity, tube patency, manufacturing reproducibility, and storage requirements. These practical constraints are especially relevant when delivering nutrition to critically ill patients via small-bore feeding tubes over prolonged periods. Consequently, future research should evaluate not only whether more food-based formulations alter biological responses, but also whether they can maintain the safety, consistency, and practicality required for widespread clinical use.

None of these practical constraints diminish the importance of studying formula composition. Rather, they illustrate why the next generation of studies should integrate mechanistic science with careful clinical evaluation. Future research should account not only for biological complexity and patient heterogeneity, but also for the practical realities of formulation design, manufacturability, and food safety. Ultimately, the challenge is not simply to identify biologically attractive hypotheses, but to determine which aspects of formula composition produce reproducible improvements in outcomes that matter to patients while remaining practical and safe for routine critical care delivery.
